# The effect of the unite of meridian flow chronotherapy and acupressure in improving sleep reversal in elderly people

**DOI:** 10.1093/geroni/igaf122.2670

**Published:** 2025-12-31

**Authors:** Jiajia Xu, Dan Liu, Shan Li, Yue Hu

**Affiliations:** Shanxi Bethune Hospital, Taiyuan, Shanxi, China (People’s Republic); Shanxi Bethune Hospital, Taiyuan, Shanxi, China (People’s Republic); Shanxi Bethune Hospital, Taiyuan, Shanxi, China (People’s Republic); Shanxi Bethune Hospital, Taiyuan, Shanxi, China (People’s Republic)

## Abstract

**Background:**

Global aging is accelerating, and the incidence of sleep reversal in the elderly has increased significantly, affecting the quality of life. The Chinese Meridian Flow Theory, which advocates the regulation of meridians and qi-blood at specific hours to restore physiological rhythms, has demonstrated advantages in the management of chronic diseases.

**Objective:**

To explore efficient and safe intervention programs to improve sleep inversion and to provide theoretical support and practical reference for preclinica.

**Methods:**

Intervention: For yin and yang imbalance and visceral disharmony, select the heart meridian, kidney meridian, pericardium meridian acupuncture points, and construct the “three time intervention axis”①11:00-13:00 Shenmen, Shaohai; ②17:00-19:00 Yongquan, Taixi; ③19: 00-21:00 Neiguan, Laogong. Each point is pressed using the fingertips for 3 minutes and circulated 3 times to localize a slight redness of the skin. Control: Routine sleep health promotion. RESULTS:The number of nocturnal awakenings (1.2±0.5 times ), time (28.5±10.2 minutes), and PSQI score (6.2±1.8 points) of the intervention group were significantly better than those of the control group (P < 0.05). The TCM evidence points were 37.5%-47.8% lower than those of the control group, and the efficacy was stable during the follow-up period without serious adverse reactions.

**Conclusion:**

This measure is effective in improving sleep regression in the elderly. It contributes Chinese medicine wisdom to the WHO Global Strategy on Aging and Health, and promotes “active and healthy” aging with the synergy of Chinese and Western medicine.

